# Histochemical and Immunohistochemical Characterizations of Hepatic Trematodiasis in Odontocetes

**DOI:** 10.3389/fvets.2020.00336

**Published:** 2020-06-30

**Authors:** Shotaro Nakagun, Yoshiyasu Kobayashi

**Affiliations:** ^1^Laboratory of Veterinary Pathology, Department of Veterinary Medicine, Obihiro University of Agriculture and Veterinary Medicine, Obihiro, Japan; ^2^United Graduate School of Veterinary Sciences, Gifu University, Gifu, Japan

**Keywords:** cetacean, liver, fluke, pathology, parasite, macrophage, *Campula oblonga*, *Oschmarinella macrorchis*

## Abstract

Hepatic trematodiasis is a common condition in a number of free-ranging cetacean species, which occasionally result in severe hepatic and/or pancreatic lesions. However, even the basic pathological information of this disease is unknown for the majority of affected species. The current study describes and compares the histomorphology and immune reaction induced by hepatic trematodes of the family Brachycladiidae in the liver of the harbor porpoise (*Phocoena phocoena, n* = 8), Dall's porpoise (*Phocoenoides dalli, n* = 8), and Hubbs' beaked whale (*Mesoplodon carlhubbsi, n* = 2). Immunohistochemistry for eight antibodies (CK19, CD3, Foxp3, CD20, Iba1, CD68, CD163, and CD204) was conducted to analyze the pathology of these parasitic infections. In all three odontocete species, the changes observed in the trematode-affected biliary epithelium were comparable with marked hyperplasia and goblet cell metaplasia, as well as lymphoplasmacytic and eosinophilic inflammation. Additionally, regions of the Glisson's sheath were diffusely and severely fibrotic in all examined species, regardless of the physical presence of trematodes. Differences among the three species included the presence of characteristic lymphoid follicles formed in the fibrotic bile duct walls of only the two porpoise species. In the Hubbs' beaked whale, the degree of lymphoplasmacytic cholangitis was more severe, and ductular reaction was generally more prominent. In terms of the overall macrophage population among the three species, CD163- and CD204-positive cells (M2 macrophages) outnumbered Iba1- and CD68-positive cells (M1 macrophages), indicating a chronic infection stage in all analyzed individuals. Species-specific differences among the infiltrating macrophages included numbers of CD68-positive cells being significantly more abundant in the harbor porpoises, whereas CD163-positive cells were significantly more numerous in the Dall's porpoises. The numbers of CD204-positive macrophages were higher in the Hubbs' beaked whales compared to those in the porpoises. Trematode species of the harbor and Dall's porpoises were *Campula oblonga*, while they were *Oschmarinella macrorchis* in the Hubbs' beaked whales. This study concludes that interspecies differences in the tissue reactions to hepatic trematode infections are present among odontocete species and that the immune reaction varies depending on the species. This information aids in furthering our understanding of the pathogenesis of hepatic trematodiasis in cetaceans.

## Introduction

Parasitic infections are one of the most common pathological findings recognized among free-ranging cetaceans (1–5). These parasites affecting cetaceans are highly diverse, ranging from non-pathogenic species such as cestodes of *Monorygma* spp. (6) and highly harmful species such as nematodes of *Crassicauda* spp. (7–9). One of the groups considered pathological in nature are trematodes that consist of four families in cetaceans, namely, the families Brachycladiidae, Heterophyidae, Brauninidae, and Notocotylidae (10). The family of most importance in terms of diversity and distribution is the Brachycladiidae (11), consisting of 52 species in which 45 impact various cetaceans across the globe (10).

Species of *Campula* and *Oschmarinella* among the family Brachycladiidae are found in the bile and pancreatic ducts of odontocetes (toothed whales), occasionally acting as problematic pathogens (12, 13). *Campula oblonga* is a species commonly infecting porpoises of the family Phocoenidae, including the harbor porpoise *Phocoena phocoena* (6, 14), Dall's porpoise *Phocoenoides dalli* (15–17), and the narrow-ridged finless porpoise *Neophocaena asiaeorientalis* (18, 19) but also occasionally infecting others like the common dolphin *Delphinus delphis* (20, 21). Histopathology of the lesions caused by *Campula* has been well-documented with hypertrophy of the biliary epithelium, portal fibrosis, cholangitis, pericholangitis, and formation of lymphoid follicles (22–24). On the other hand, *Oschmarinella macrorchis* has been reported only from the biliary tracts of Stejneger's beaked whales *Mesoplodon stejnegeri* (25) and Hubbs' beaked whales *Mesoplodon carlhubbsi* (26), indicating a narrower host range. Descriptions of *Oschmarinella*-infected liver histopathology have been scarce to date with limited reports of fibrosis and pericholangitis (12, 27, 28) and a single case report from a Hubbs' beaked whale describing prominent ductular reaction and lack of lymphoid follicles (26). The lesions described in the Hubbs' beaked whale had not been described in hepatic trematode infections of marine mammals before, indicating a possibility of interspecies difference in the response against various trematode species.

Hepatic fibrosis is a healing response to various chronic liver injuries such as viral infections and cholestasis (29). It is caused by a combination of inflammation and activation of stellate cells in which it triggers an accumulation of extracellular matrix, and among other complex mechanisms, the role of macrophages including Kuppfer cells are considered important (30, 31). In the case of hepatic trematodiasis in cetaceans, severe fibrosis is considered to be due to the physical presence of the parasite within bile ducts and exfoliated eggs around portal tracts (13). There have been several reports on the infiltrating inflammatory cell types (13, 32, 33), but details of the infiltrating and resident macrophages and the host's immune response have not been investigated.

The current study focused on the comparative immune responses of the harbor porpoise, Dall's porpoise, and Hubbs' beaked whale against hepatic trematode infections by characterizing the histomorphology as well as immunophenotyping the inflammatory cell population and tissue macrophages.

## Materials and Methods

### Study Population

The formalin-fixed paraffin embedded (FFPE) liver tissues used in this study were obtained from eight harbor porpoises (case nos. 1–8), eight Dall's porpoises (case nos. 9–16), and two Hubbs' beaked whales (case nos. 17–18) of various sex and age class (34) from Hokkaido, northern Japan, between 2013 and 2019 (Table 1). The numbers of females and males were equal for the harbor porpoise (body lengths, 127.2–186.5 cm) and Hubbs' beaked whales (body lengths, 493.0, 510.0 cm), while there were three females and five males for the Dall's porpoises (body lengths 169.1–204.3 cm). Cases were obtained from naturally stranded animals except for three harbor porpoises and two Dall's porpoises, which died of fishing gear entanglement. The specimens were selected on the basis of decomposition codes (35) to guarantee detailed histopathological and immunohistochemical evaluations. Detailed gross and histopathological investigations were performed on all of these cetaceans, confirming that they were free of any severe systemic and/or liver specific diseases other than the liver fluke infection, which may potentially alter the hepatic histomorphology.

**Table 1:** Biological and parasitological data of the examined harbor porpoises, Dall's porpoises, and Hubbs' beaked whales (*Mesoplodon carlhubbsi*).

**Case No**.	**Case ID**	**Species**	**Date found**	**Stranding site**	**Sex**	**BL (cm)**	**Age**	**DC**	**Hepatic trematode species**
1	SNH13012	Harbor porpoise	05/17/13	43°57′52.0″N, 145°08′25.7″E	M	131.0	SA	2	*Campula oblonga*
2	SNH16011-2	Harbor porpoise	04/26/16	41°56′37.8″N, 140°58′01.7″E	M	127.2	SA	2	*C. oblonga*
3	SNH16023	Harbor porpoise	07/16/16	42°46′38.6″N, 143°46′47.6″E	F	186.5	A	2	*C. oblonga*
4	SNH16031	Harbor porpoise	09/06/16	41°56′19.8″N, 143°12′34.9″E	M	138.3	SA	2	*C. oblonga*
5	SNH17003	Harbor porpoise	02/24/17	43°12′54.7″N, 140°55′16.1″E	M	139.5	SA	2	*C. oblonga*
6	SNH17005	Harbor porpoise	04/02/17	42°37′26.4″N, 141°35′03.7″E	F	133.1	SA	2	*C. oblonga*
7	SNH17007	Harbor porpoise	04/04/17	42°37′07.3″N, 141°33′27.0″E	F	138.6	SA	2	*C. oblonga*
8	SNH18012	Harbor porpoise	04/25/18	41°56′37.8″N, 140°58′01.7″E	F	152.2	A	2	*C. oblonga*
9	SNH14034-1	Dall's porpoise	07/04/14	43°52′36.8″N, 145°08′39.0″E	M	193.5	SA	2	*C. oblonga*
10	SNH14034-3	Dall's porpoise	07/04/14	43°52′36.8″N, 145°08′39.0″E	M	198.6	SA	2	*C. oblonga*
11	SNH19003-1	Dall's porpoise	02/07/19	44°02′59.6″N, 145°13′54.3″E	M	190.4	SA	2	*C. oblonga*
12	SNH19003-2	Dall's porpoise	02/07/19	44°02′59.6″N, 145°13′54.3″E	F	176.9	SA	2	*C. oblonga*
13	SNH19003-5	Dall's porpoise	02/07/19	44°02′59.6″N, 145°13′54.3″E	F	176.5	SA	2	*C. oblonga*
14	SNH19003-7	Dall's porpoise	02/07/19	44°02′59.6″N, 145°13′54.3″E	M	185.8	SA	2	*C. oblonga*
15	SNH19003-10	Dall's porpoise	02/07/19	44°02′59.6″N, 145°13′54.3″E	F	169.1	J	2	*C. oblonga*
16	SNH19003-11	Dall's porpoise	02/07/19	44°02′59.6″N, 145°13′54.3″E	M	204.3	A	2	*C. oblonga*
17	SNH15011	Hubbs' beaked whale	04/14/15	42°07′21.4″N, 142°56′56.3″E	M	493.0	A	2	*Oschmarinella macrorchis*
18	SNH18034	Hubbs' beaked whale	08/31/18	42°56′32.0″N, 144°28′30.4″E	F	510.0	A	3	*O. macrorchis*

*F, female; M, male; BL, body length; age, age class (A, adult; SA, subadult; J, juvenile); DC, decomposition code (2, minimal autolysis; 3, moderate autolysis); SNH, stranding network hokkaido*.

### Histopathology, Histochemistry, and Immunohistochemistry

One FFPE liver block with the most prominent of trematode lesions was selected per animal for histopathological analyses. Serial sections were made from each block at 4 μm, and hematoxylin and eosin (HE) sections were prepared for review of general histomorphology. The histochemical stains used and their purposes were as follows: Masson's trichrome, to highlight the presence and distribution of reactive fibrosis; reticulin, to assess the hepatic plate architecture and regeneration; and Prussian blue, to demonstrate intracellular iron and evaluate hepatocellular injury. The extent of lymphoid follicle formation, periportal and perivenular/perisinusoidal fibrosis, ductular reaction, and iron deposition in the Kuppfer cells and hepatocytes were graded semiquantitatively as none (0), minimal (1), mild (2), moderate (3), and severe (4).

For immunohistochemistry, the following eight primary antibodies were applied: cytokeratin (CK) 19, cluster of differentiation (CD) 3, forkhead box protein P3 (Foxp3), CD20, ionized calcium binding adaptor molecule 1 (Iba1), CD68, CD163, and CD204. Details on the antibodies and protocol are listed in Table 2. Briefly, antigen retrieval was followed by blocking of endogenous peroxidase activity with 0.3% hydrogen peroxide incubation for 10 min at room temperature, and blocking of non-specific staining with 5% skim milk for 45 min at room temperature. The slides with each primary antibody were incubated overnight at 4°C in a humidified chamber. Antigen visualization was performed using the Histofine Simple Stain MAX-PO kit (Nichirei Biosciences, Tokyo, Japan), followed by 3,3′-diaminobenzidine (Nichirei Biosciences) as chromogen, with a Meyer's hematoxylin counterstain. In place of the MAX-PO kit, the protocol for Foxp3 differed by a sequential incubation with a biotin-labeled antirat immunoglobulin (IgG) antibody (1:200; Vector Laboratories, California, USA) and a horseradish peroxidase-conjugated streptavidin (1:400; Dako, Glostrup, Denmark) at room temperature for 30 min, respectively. Tissue sections in which the primary antibody was replaced by non-immune mouse/rabbit/rat serum served as negative controls, respectively, while normal livers and lymph nodes of the harbor porpoise/Dall's porpoise/Hubbs' beaked whale were used as positive controls.

**Table 2:** Details of the primary antibodies tested for immunohistochemistry with working dilutions and antigen retrieval methods.

**Antigen**	**Clone**	**Species**	**Dilution**	**Antigen retrieval**	**Manufacturer**	**Specificity**
CD3	F7.2.38	Mouse	1:100	Microwave^a^	Dako, Glostrup, Denmark	T cell
Foxp3	FJK-16S	Rat	1:200	Autoclave^b^	eBioscience, California, USA	Regulatory T cell
CD20	–	Rabbit	1:800	Microwave	Thermo Fisher, California, USA	B cell
Iba1	–	Rabbit	1:1,000	Microwave	Wako, Osaka, Japan	Macrophage
CD68	EBM11	Mouse	1:50	Proteinase K^c^	Dako	Exudate macrophage
CD163	AM-3K	Mouse	1:50	Proteinase K	Trans Genic, Kumamoto, Japan	Kuppfer cell
CD204	SRA-E5	Mouse	1:30	Microwave	Trans Genic	Macrophage, Kupffer cell
CK19	–	Rabbit	1:200	Microwave	Abcam, Cambridge, UK	Cholangiocyte

*CD, cluster of differentiation; Foxp3, forkhead box protein P3; Iba1, ionized calcium binding adaptor molecule 1; CK, cytokeratin*.

a *Sodium citrate buffer pH 6.0 in microwave at 97°C for 15 min*.

b *Sodium citrate buffer pH 6.0 in autoclave at 121°C for 20 min*.

c *0.02% proteinase K at 37°C for 45 min*.

### Cell Counts and Statistical Analysis

Photomicrographs of five randomly selected areas at the lamina propria of the proliferative bile duct epithelium (BD), portal fibrotic tissue including lymphoid follicles (PF), and hepatic parenchyma adjacent to the portal fibrotic tissue (HP) in each animal were taken at a magnification of 400× for each of the CK19, CD3, Foxp3, CD20, Iba1, CD68, CD163, and CD204 stained samples. For CD3, Foxp3, and CD20, photomicrographs of only the BD and PF areas were analyzed due to the consistently minimal number of immunopositive cells in the hepatic parenchyma. Additionally, the extent of biliary ductular reaction was evaluated on a scale of 1–4 with the number of immunoreactive cells for CK19 in the PF areas (1 = <30; 2 = 30–60; 3 = 61–100; 4 = 101 <). The numbers of distinctively immunopositive cells for each area (0.036 mm^2^) were counted using the ImageJ software version 1.52q (National Institutes of Health, MD, USA), and the mean values for each area per antigen and animal were obtained. In order to compare the interspecies differences in the immune response, the median, mean, and standard deviation for each of the species were calculated per area and antigen, and statistical differences among the harbor porpoises and Dall's porpoises were determined by the two-sided Mann–Whitney *U*-test. Difference with *P* < 0.05 was considered significant. Data from the Hubbs' beaked whales were not included in the statistical test since the number of infected animals was too small at *n* = 2.

### Trematode Identification

At the time of postmortem examinations of each animal, the hepatic trematodes were subjected to simplified morphological observations. Genomic DNA of the trematodes were extracted either from specimens fixed and stored in 100% ethanol or from FFPE liver tissue containing the parasite, using the NucleoSpin Tissue kit (MACHEREY-NAGEL, Düren, Germany) following the manufacturer's instructions. PCR and subsequent sequencing of the trematode mitochondrial DNA NADH dehydrogenase subunit 3 (mtND3) sequence was conducted using primers ND3F (36) and ND3-4 (37) as described previously (26). The obtained DNA sequences were analyzed on Sequence Scanner Software, version 2 (Applied Biosystems, California, USA), and aligned on MEGA7, version 7.0.18 with the MUSCLE program. The phylogenetic relationships of the obtained sequences were analyzed with a known *C. oblonga* sequence obtained from the Baltic Sea (GenBank accession no. AF034554) and a previously identified *O. macrorchis* sequence of Case no. 18 (GenBank accession no. LC326064). *Tormopsolus orientalis* (GenBank accession no. KT180219) was set as the outgroup following formerly described relationships (38).

## Results

### Histopathological Characterization

The results for the semiquantitative histomorphological and histochemical analyses are summarized in Table 3 and Supplementary Table 1.

**Table 3:** Summary of the results for semiquantitative analyses of various histopathological features in hepatic trematode infected livers of the harbor porpoise, Dall's porpoise, and Hubbs' beaked whale.

**Species**		**Lymphoid follicle**	**Periportal fibrosis**	**Bridging fibrosis**	**Iron deposition (hepatocyte)**	**Iron deposition (Kuppfer cell)**	**Ductular reaction**
Harbor porpoise	Median	3.0	4.0	3.0	0.0	3.0	3.0
	Mean	3.0	3.8	2.9	0.3	2.5	2.9
	SD	1.1	0.5	1.2	0.7	0.8	0.6
Dall's porpoise	Median	3.0	4.0	3.0	0.0	2.0	2.5
	Mean	2.9	3.9	3.3	0.1	2.1	2.9
	SD	1.1	0.4	0.7	0.4	0.8	1.0
Hubbs' beaked whale	Median/Mean	1.0	4.0	3.5	0.5	1.5	4.0
	SD	0	0	0.7	0.7	2.1	0

*Grading scale: 0, none; 1, minimal; 2, mild; 3, moderate; 4, severe*.

**Figure 1:**
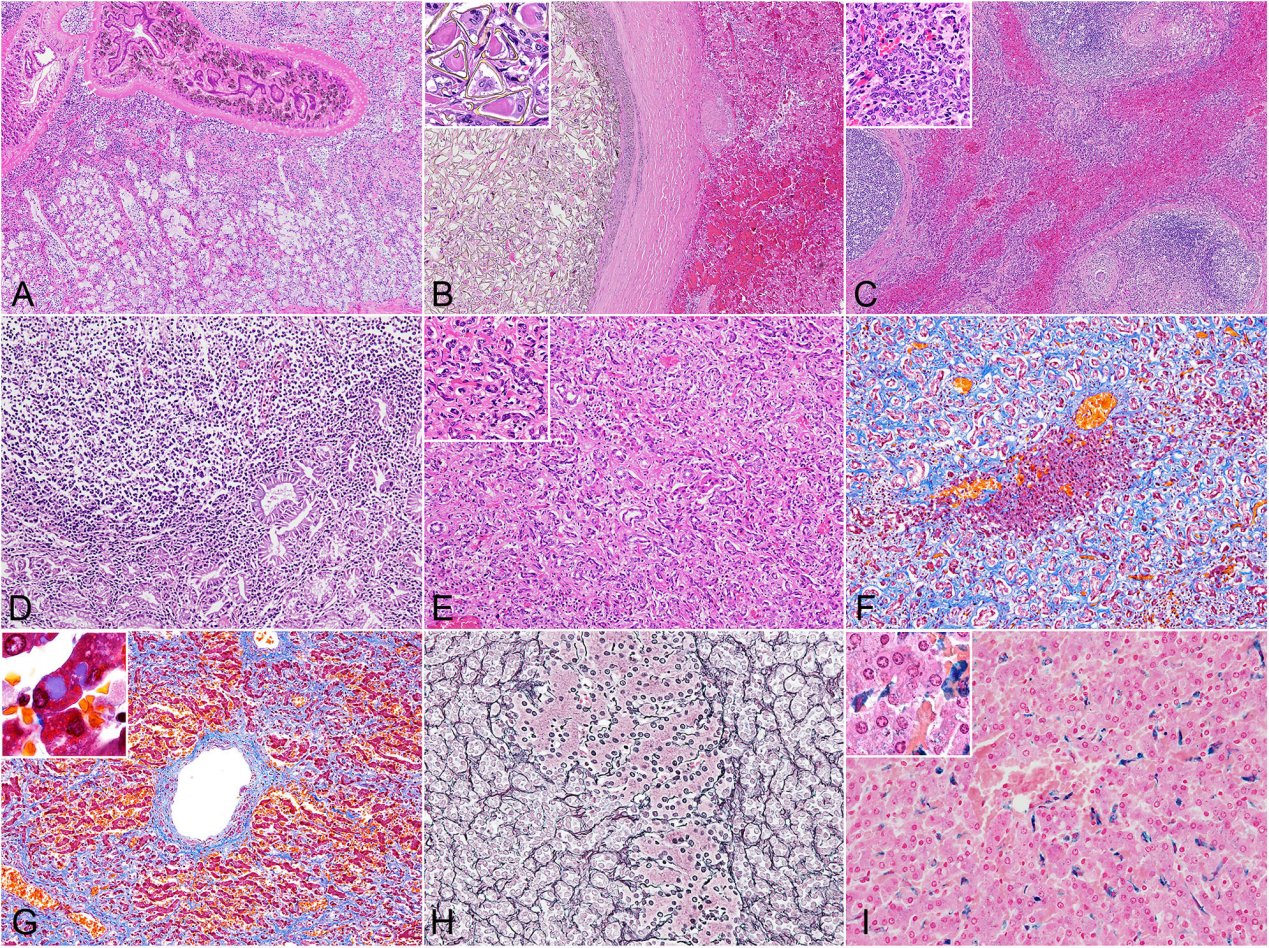
Histopathology of hepatic trematodiasis, liver, odontocetes. **(A)** Trematodes are present within the dilated biliary lumen, while the biliary epithelium is highly hyperplastic. Case no. 3, harbor porpoise. Hematoxylin and eosin (HE). **(B)** Numerous trematode eggs occlude the dilated biliary lumen. Note hemorrhage in the adjacent parenchyma. Inset, higher magnification of triangular-shaped eggs within the lumen. Case no. 9, Dall's porpoise. HE. **(C)** Large lymphoid follicles are formed in portal areas, while abundant bridging fibrosis dissects normal hepatic architecture. Inset, higher magnification of the reactive biliary epithelium in areas of bridging fibrosis. Case no. 4, harbor porpoise. HE. **(D)** Severe lymphoplasmacytic infiltration in the lamina propria of a dilated bile duct, partially effacing the hyperplastic biliary epithelium. Case no. 17, Hubbs' beaked whale. HE. **(E)** Severe ductular reaction replaces the hepatic parenchyma. Inset, higher magnification of proliferating bile ducts. Case no. 17, Hubbs' beaked whale. HE. **(F)** An island of hepatocytes remains within an area of prominent ductular reaction with fibrosis. Case no. 17, Hubbs' beaked whale. Masson's trichrome. **(G)** Individuals with severe fibrosis are characterized with portal-to-portal bridging fibrosis, perivenular and perisinusoidal fibrosis, and a fibrous septum formation. Inset, demonstration of intracytoplasmic inclusion-like vacuoles in hepatocytes. Case no. 13, Dall's porpoise. Masson's trichrome. **(H)** An island of hepatocytes is not delineated by apparent fibrous scarring, and the transition from hepatocytes to proliferative bile ducts is uninterrupted by reticular fibers, indicating a lack of apparent hepatocellular damage. Note the relatively organized hepatocyte cords. Case no. 12, Dall's porpoise. Reticulin. **(I)** Moderate amounts of Prussian blue-positive hemosiderin deposits are detected in Kuppfer cells. Inset, demonstration of intracytoplasmic hemosiderin also within hepatocytes. Case no. 2, harbor porpoise. Prussian blue.

The histomorphology of the bile duct epithelium were similar in all three species. Within the severely dilated bile ducts were trematodes with suckers and numerous cuticles on the body surface (Figure 1A). These parasites were surrounded by a mixed inflammatory population of eosinophils, lymphocytes, plasma cells, and macrophages, along with infrequent triangular trematode eggs and moderate amounts of cellular debris. The biliary epithelium was highly hyperplastic with goblet cell metaplasia, and the lamina propria contained mild to severe infiltrates of inflammatory cells as described. Inflammation was especially severe in the Hubbs' beaked whales, while small lymphoid follicles were formed in some of the porpoises. In two of the Dall's porpoises (case nos. 9 and 10) were cystic and embolic bile ducts filled with abundant trematode eggs (Figure 1B). The adjacent hepatic parenchyma in these areas was hemorrhagic.

The walls of the dilated bile ducts in the harbor porpoises and Dall's porpoises were thickened due to moderate to severe fibrosis. In these walls were frequent formations of large lymphoid follicles, focal to locally extensive inflammation, and mild to moderate ductular reaction. Similar changes were observed in the Glisson's sheath, with fibrosis, eosinophil-rich inflammation, formation of small lymphoid follicles, and mild biliary hyperplasia. In animals with severe fibrosis, bridging fibrosis with ductular reaction expanded the Glisson's sheaths (Figure 1C). However, the degree of fibrosis in the portal region and level of bridging fibrosis did not necessarily correlate to each other. The levels of bridging fibrosis and ductular reaction were more severe in the harbor porpoises compared to the Dall's porpoises. Small granulomas in reaction to trematode eggs were observed in the portal regions of all animals except for two harbor porpoises (case nos. 3 and 7) and one Dall's porpoise (case no. 16).

The histological reaction in the Hubbs' beaked whales was unique compared to the porpoises by the following three changes. First, the lamina propria of the hyperplastic biliary epithelium was severely infiltrated by lymphocytes and plasma cells, resulting in a lymphoplasmacytic cholangitis (Figure 1D). Secondly, instead of mature lymphoid follicles forming in the fibrotic bile duct walls, there were small and loose aggregates of mononuclear cells, primarily composed of lymphocytes. Finally, severe fibrosis and ductular reaction not only affected the Glisson's sheaths but also the adjacent parenchyma (Figure 1E).

Findings from histochemical stains were as follows: the Masson's trichrome stain highlighted collagen fibers of periportal and bridging fibrosis. In individuals with severe fibrosis, there was diffuse loss of hepatic parenchyma (Figure 1F), and scarring around the central vein extended to portal areas, forming a fibrous septum (Figure 1G). Additionally, some of the periportal scar tissue stained lighter compared to collagen fibers of the portal tract. With the reticulin stain, regenerative nodules were not observed in any of the individuals. Transition from hepatocytes to cholangiocytes was smooth between small foci of hepatocytes and surrounding advanced ductular reaction, and the reticulin fibers within the remaining hepatocyte cords were organized (Figure 1H). In the Hubbs' beaked whales, postmortem changes obscured detailed structures of hepatocytes and reticulin fibers. The Prussian blue stain demonstrated intracellular iron deposits within hepatocytes in only a single animal from each species, where these individuals had severe periportal fibrosis. Minimal to moderate iron deposits within Kuppfer cells (Figure 1I) were noted in every animal except for one Hubbs' beaked whale.

Changes in hepatocytes included cytoplasmic vacuoles of various sizes in the majority of individuals. These vacuoles were slightly eosinophilic on the HE stain and stained blue tinged with purple on the Masson's trichrome stain (Figure 1G inset). Some of the animals had mild to moderate cytoplasmic lipofuscin deposits.

Moderate to severe pancreatic lesions were found in 2/8 harbor porpoises and 6/8 Dall's porpoises with intralesional trematodes of the same species (Supplementary Figure 1). These lesions were comparable to that of the liver, characterized by ductal hyperplasia, eosinophil-rich inflammation, and fibrosis of the duct wall and adjacent parenchyma with formation of numerous lymphoid follicles. In the pancreas of a harbor porpoise (case no. 8) and a Dall's porpoise (case no. 11) were granulomatous cysts of up to 4 cm in diameter, containing thick dark-green material admixed with adult trematodes and numerous eggs. Slight ductal hyperplasia was detected in four harbor porpoises and two Dall's porpoises that had no trematodes present in the ducts.

### Quantitative Analyses and Immunophenotyping of Infiltrative Cells

Cross-reactivity to all antibodies was confirmed. These immunohistochemical results are summarized in Table 4 and Supplementary Table 2.

**Table 4:** Summary of the results for selected immunomarkers in hepatic trematode infected livers of the harbor porpoise, Dall's porpoise, and Hubbs' beaked whale.

		**CK19**	**CD3**		**Foxp3**		**CD20**		**Iba1**			**CD68**			**CD163**			**CD204**		
**Species**		**PF**	**BD**	**PF**	**BD**	**PF**	**BD**	**PF**	**BD**	**PF**	**HP**	**BD**	**PF**	**HP**	**BD**	**PF**	**HP**	**BD**	**PF**	**HP**
Harbor porpoise	Median	76.9	25.4	88.3	2.1	6.0	31.5	304.5	29.1	42.5	17.9	2.9	5.1^*^	5.2^†^	1.3	0.6^‡^	19.2	4.9	2.9	18.9
	Mean	75.7	26.8	81.3	2.7	5.2	36.4	272.3	33.5	43.2	16.0	3.8	5.6	5.9	1.9	0.7	20.9	8.1	9.7	20.4
	SD	22.4	23.3	57.6	1.8	3.0	20.9	113.1	21.7	24.5	8.5	2.9	4.6	4.1	1.8	0.4	6.1	10.3	11.6	10.4
Dall's porpoise	Median	71.2	22.6	92.0	2.8	6.2	27.6	304.6	18.0	34.3	14.3	1.4	1.2^*^	1.2^†^	3.6	4.4^‡^	21.1	22.6	12.6	22.5
	Mean	97.1	26.4	102.3	3.5	6.1	31.6	274.0	19.5	40.1	14.4	2.1	1.3	1.6	8.7	7.0	22.0	20.3	11.7	21.6
	SD	55.7	20.7	66.1	4.2	3.8	19.6	109.0	5.4	21.2	9.6	2.0	0.7	1.4	9.0	7.5	7.6	9.0	7.1	5.8
Hubbs' beaked whale	Median/Mean	125.3	3.7	53.8	1.1	1.3	92.7	81.1	15.9	36.9	1.9	0.3	1.0	0.3	1.9	20.7	22.7	31.4	57.1	50.4
	SD	10.9	1.0	58.3	1.3	1.6	40.3	65.8	5.5	10.3	1.3	0.1	0.6	0.1	2.4	29.0	23.6	2.0	41.7	4.5

*BD, lamina propria of proliferative bile duct; PF, portal fibrotic tissue including lymphoid follicles; HP, periportal hepatic parenchyma; SD, standard deviation; NA, not assessed*.

* *Significant difference at P = 0.014*.

† *Significant difference at P = 0.036*.

‡ *Significant difference at P = 0.005*.

**Figure 2:**
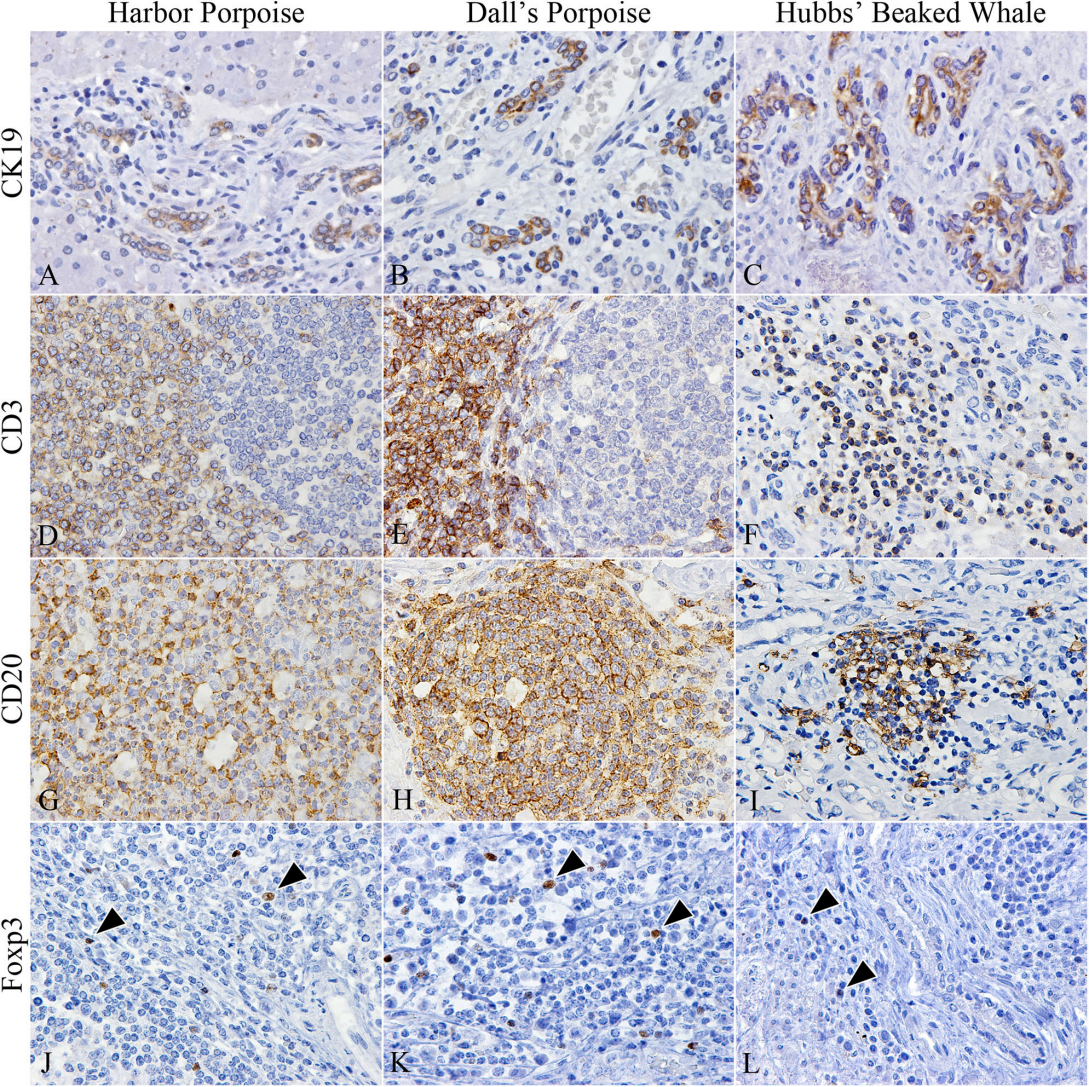
Characterization of cholangiocytes and inflammatory cell populations in hepatic trematode infected livers of the harbor porpoise (left), Dall's porpoise (center), and Hubbs' beaked whale (right). **(A–C)** CK19-positive cholangiocytes are detected in areas of fibrosis, which form irregularly shaped hyperplastic bile ducts (**A**, case no. 3; **B**, case no. 14; **C**, case no. 18). **(D–F)** CD3-positive T cells are found in the paracortical areas of formed lymphoid follicles in the harbor porpoise (**D**, case no. 2) and Dall's porpoise (**E**, case no. 12), while it is more diffusely scattered within inflammatory infiltrates in the Hubbs' beaked whale (**F**, case no. 17). **(G–I)** Numerous CD20-positive B cells are detected within formed lymphoid follicles in the harbor porpoise (**G**, case no. 7) and Dall's porpoise (**H**, case no. 9), while the inflammatory infiltrates in the Hubbs' beaked whales have a mixed cell population (**I**, case no. 17). **(J–L)** Low numbers of Foxp3-positive regulatory T cells (arrowheads) are scattered within the inflammatory infiltrates of all three species (**J**, case no. 4; **K**, case no. 15; **L**, case no. 18).

The number of CK19-positive cholangiocytes varied greatly among individuals. The average numbers of cholangiocytes were greatest in the order of Dall's porpoises, Hubbs' beaked whales, and harbor porpoises (Figures 2A–C), although the extent of ductular reaction was most prominent in the Hubbs' beaked whale.

CD3-positive T and CD20-positive B cells were abundant in the porpoises, which had frequent lymphoid follicles. These formed follicles were histologically indifferent from normal follicles in lymphoid organs primarily composed of B cells, and there were no interspecies differences among the numbers of T and B cells. Whereas, in the Hubbs' beaked whales, the inflammatory foci consisted of an even mixture of T and B cells (Figures 2D–I), in the BD region of the Hubbs' beaked whale, there was marked and diffuse infiltration of B cells. Foxp3-positive regulatory T cells were limited in all analyzed individuals, while comparatively large numbers were detected within inflammatory foci and lymphoid follicles of BD and PF regions, respectively (Figures 2J–L).

**Figure 3:**
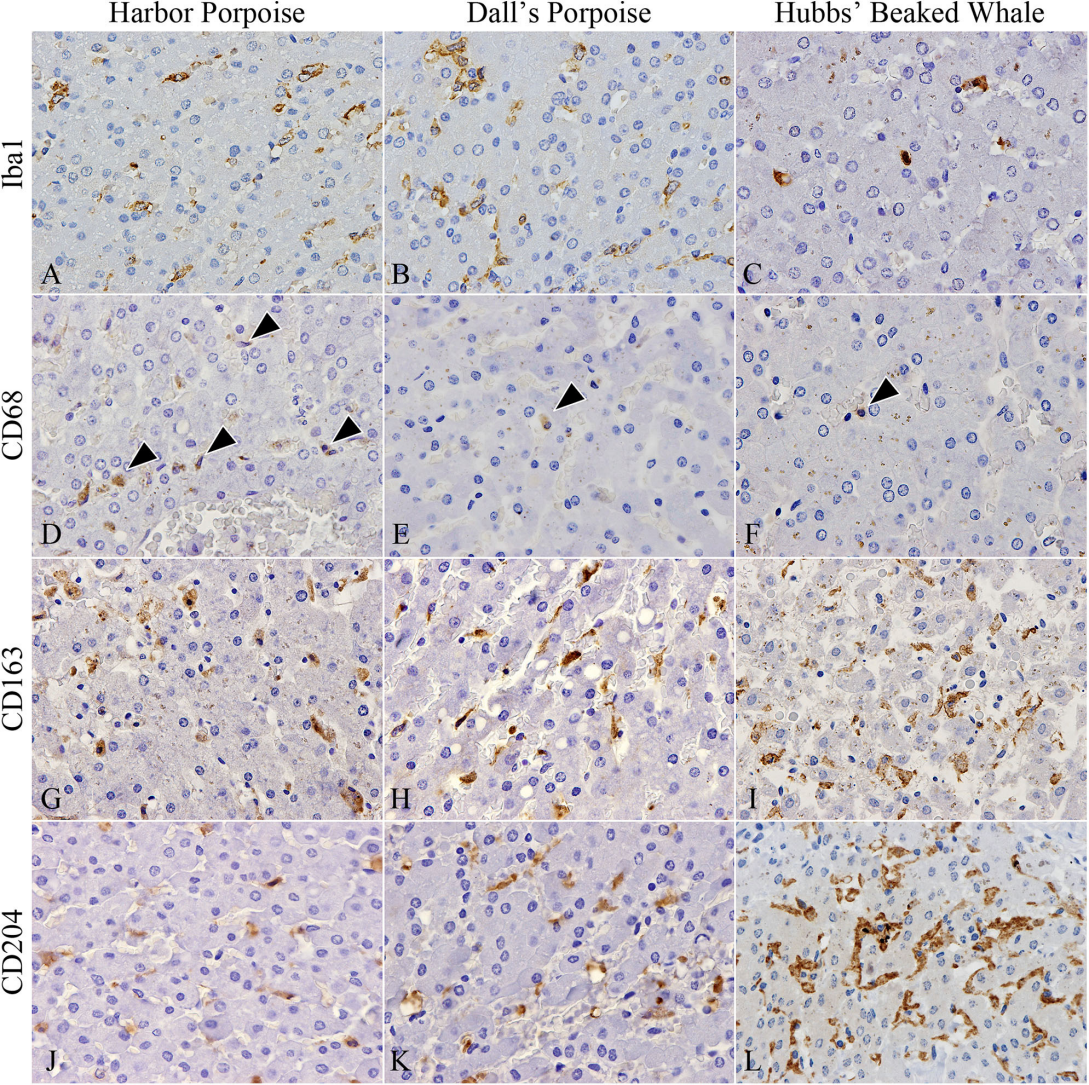
Distribution of various macrophages in hepatic trematode infected livers of the harbor porpoise (left), Dall's porpoise (center), and Hubbs' beaked whale (right). **(A**–**C)** Iba1-positive macrophages aggregate in moderate numbers within areas of the periportal hepatic parenchyma. (**A**, case no. 1; **B**, case no. 12; **C**, case no. 17). **(D–F)** CD68-positive macrophages (arrowheads) are infrequent in all three species (**D**, case no. 2; **E**, case no. 11; **F**, case no. 17). **(G–I)** CD163-positive Kuppfer cells in the periportal hepatic parenchyma are found in close numbers across all three species (**G**, case no. 3; **H**, case no. 15; **I**, case no. 18). **(J–L)** CD204-positive Kuppfer cells in the periportal hepatic parenchyma appear to be most abundant in the Hubbs' beaked whale (**J**, case no. 6; **K**, case no. 13; **L**, case no. 17).

In terms of macrophage immunophenotyping, relatively large numbers of Iba1-positive cells were observed in inflammatory foci, while they were generally limited in the HP regions (Figures 3A–C). Although there were no apparent interspecies differences, the greatest number was recorded in the BD region of the harbor porpoises. CD68-positive cells were the least recorded macrophage type in all species, where they were better observed in the harbor porpoises (Figures 3D–F). The number of CD163-positive cells were increased in the HP region, staining Kuppfer cells (Figures 3G–I). Similarly, CD204-positive cells were most plentiful in the HP region (Figures 3J–L), although large numbers were also observed in the BD and PF regions for some individuals. The Hubbs' beaked whales tended to have more CD204-positive cells compared to the two porpoise species, where apparent disparities in the average positive cells numbers were recorded particularly in the PF and HP regions.

Significant differences among the harbor and Dall's porpoises were calculated for CD68-positive cells in the PF and HP regions (*P* = 0.014, *P* = 0.036, respectively), and for CD163-positive cells in the PF region (*P* = 0.005), where CD68-expressing cells were significantly greater in the harbor porpoises, and CD163-expressing cells in the Dall's porpoises.

### Trematode Identification

**Figure 4:**
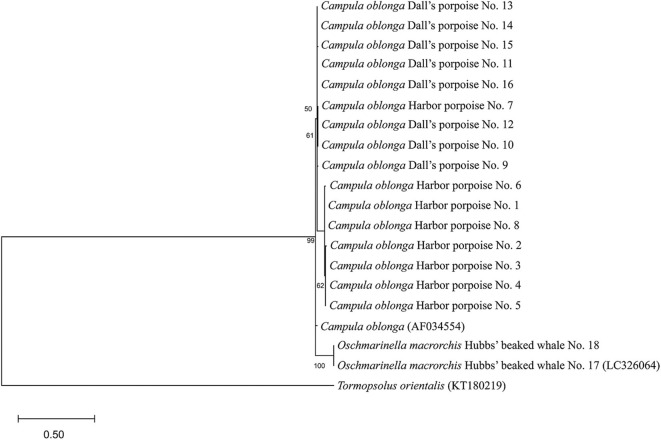
Phylogenetic relationships of Brachycladiidae trematodes *Campula oblonga* and *Oschmarinella macrorchis* based on mitochondrial DNA NADH dehydrogenase subunit 3 (mtND3) gene sequences, as inferred by the maximum likelihood method and Hasegawa–Kishino–Yano model. Bootstrap values are shown on the left of the supported node. The tree is drawn to scale, with branch lengths measured in the number of substitutions per site. Accession numbers are indicated in parentheses.

Phylogenetic analysis of the obtained mtND3 gene sequences (344 bp) revealed all trematodes of the harbor porpoises and Dall's porpoises clustering with *C. oblonga* from the Baltic Sea, while clearly separating from sequences from the Hubbs' beaked whales (Figure 4). The sequences generated from the trematodes of the two Hubbs' beaked whales were identical. Thus, trematodes of the harbor porpoises and Dall's porpoises were identified as *C. oblonga*, and trematodes of the Hubbs' beaked whales as *O. macrorchis*. Results of the molecular analyses matched with the morphological observations.

From the 16 *C. oblonga* trematodes analyzed, eight distinct haplotypes were identified. Seven of these haplotypes were found only in the same host species, but one was shared among both the harbor (*n* = 1) and Dall's (*n* = 2) porpoises. Differences of 7–21 bases were identified between *C. oblonga* of the Baltic Sea, where *C. oblonga* of the Dall's porpoises were genetically closer than those of the harbor porpoises. The novel gene sequences obtained are deposited in the GenBank/DDBJ database under accession nos. LC532144–LC532151.

## Discussion

The current study identified histopathological differences among the harbor porpoises, Dall's porpoises, and Hubbs' beaked whales infected by hepatic trematodes of *C. oblonga* and *O. macrorchis*. Despite the histomorphology in the harbor and Dall's porpoises being comparable, there were significant differences in the population of tissue macrophages. On the other hand, the Hubbs' beaked whales had a distinct immune response in comparison to the porpoise species, especially with the number of CD204-positive macrophages. Different strains of laboratory animals such as rats are known to react differently toward the same stimulant (39), and hence, interspecies differences among these cetaceans were to be expected. We conclude that the immune reactions toward hepatic trematodes in cetaceans are not uniform and are variable among species.

The histomorphology of cetacean hepatic trematodiasis has been described best in those of *Campula* sp. infection (22–24), and the findings from the harbor and Dall's porpoises were generally in accordance with these reports. There were no apparent disparities in the histomorphology between the two species, but significant differences were noted in the immunophenotype of macrophages. Moreover, the cystic bile ducts due to egg embolism observed in two of the Dall's porpoises are previously unreported. In humans and laboratory animals, obstruction of bile ducts leading to chronic cholestasis and eventual cirrhosis is a well-known pathway of hepatic failure (40). Concerns alike may be relevant in cetaceans too, when these obstructive lesions are diffusely distributed. Meanwhile, similar lesions were observed in the pancreas of the two porpoise species. Interestingly, although these porpoises have a similar immune reaction and are infected by the same trematode, *C. oblonga*, the Dall's porpoises had a higher percentage (75%) of adult trematodes being observed in the pancreatic duct compared to 25% in the harbor porpoises. Since trematode-related lesions in the pancreas occupy a relatively larger area than in the liver, severe pancreatic lesions caused by the trematodes may lead to decreased pancreatic function and eventually alimentary dysfunction, as proposed previously (12).

The immune response to hepatic trematodes in the Hubbs' beaked whale was unique, characterized by a lack of lymphoid follicles, marked lymphocytic (B cell) cholangitis, and prominent proliferation of cholangiocytes. Despite some variability in the degree of fibrosis between the two individuals analyzed, severe ductular reaction in the Glisson's sheath appears to be a characteristic feature in this species.

With the present study, there is a possibility that the interspecies differences observed in the reactions of respective species may have been due to differences in the infecting trematode species: *C. oblonga* in the harbor and Dall's porpoises and *O. macrorchis* in the Hubbs' beaked whales. However, the two trematode species only differ morphologically by the presence of the lateral diverticula and are genetically very close (26). In fact, the genetic distance inferred from its mtND3 sequences between *O. macrorchis* and *C. oblonga* are closer than that between *O. rochebruni* within the same genus (26). Therefore, from both morphological and genetic perspectives, we presumed that the host response should naturally be alike. On these accounts, the differences in the histopathology and immune response between the porpoises and the Hubbs' beaked whales were more likely to be related to the difference in host species than to the infecting parasite species.

Aside from the Hubbs' beaked whale, severe fibrosis associated with hepatic trematodiasis among cetaceans has been reported from four harbor porpoises and a striped dolphin, and they have been described as biliary cirrhosis (13). With the Masson's trichrome stain in this study, individuals with severe fibrosis had collagen fibers with a light blue coloration in addition to the dark blue fibers. Since the trichrome stain can differentiate chronic fibrosis that stains dark blue from newer fibrosis that stains lighter/grayer (41), the pathogenesis of hepatic trematodiasis in cetaceans was confirmed to be in constant progress, even in cases of chronic infection. Although none of the individuals analyzed at this time fit with the diagnosis of biliary cirrhosis, cirrhosis is most likely to be the end-stage picture of the disease. On the other hand, the same previous study has reported nodular regeneration of the hepatic tissue in animals diagnosed as biliary cirrhosis (13), which was also not present in this study. The reticulin fibers of the hepatocyte cords were maintained even within the small aggregates of hepatocytes among areas of severe ductular reaction, and hepatocellular damage appeared minimal. Furthermore, the Prussian blue stain did not show high levels of cytoplasmic iron deposition in hepatocytes of any of the individuals. In humans and rats, infection with the hepatic trematode *Clonorchis sinensis* causes excessive iron deposition in hepatocytes, and a correlation between iron deposition and the degree of liver injury has been demonstrated (42). Thus, neither hepatocellular damage nor iron deposition is heavily involved in the pathogenesis of hepatic trematodiasis in cetaceans.

To date, several immunopathological investigations have been conducted in parasite infections of cetaceans (13, 32, 33, 43, 44), but none of them has focused on macrophages in detail. Macrophages are phagocytic, antigen-presenting cells that are consistently present in tissues and are involved in maintaining tissue homeostasis (45). Macrophages migrating to lesion sites are classified into two main types: M1 and M2 macrophages. M1 macrophages are induced in the early stage of inflammation and show high phagocytic capacities while M2 macrophages are involved in tissue repair by inducing fibrosis (46). These macrophages express different antigens, where Iba1-, CD68-, and major histocompatibility complex (MHC) class II-positive cells are considered M1, and CD163- and CD204-positive cells are considered M2 macrophages (46). In the current analyses, M2 macrophages were dominant over M1 macrophages especially in the HP regions, suggesting that all analyzed individuals were in the chronic stage of fibrosis rather than in the early stage of infection and injury. The obtained number of Iba1-positive macrophages was in concordance with a previous study on the immune response of hepatic trematodiasis reporting limited numbers of MHC class II-positive macrophages (33). On the contrary, an investigation of fascioliasis with *Fasciola hepatica* in cattle *Bos taurus*, an evolutionarily related species to cetaceans, had approximately equal numbers of M1 and M2 macrophages (47), and hence, differences were recognized between responses of cetaceans and cattle to hepatic trematodes.

The stage of disease was also inferred from the number of Foxp3-positive cells. In the three species analyzed, while Foxp3-positive cells were found in the lesion sites of all individuals, the numbers were generally small. Experimental infection of *F. hepatica* in sheep *Ovis aries* have demonstrated that the number of regulatory T cells is significantly higher in the early stages of infection and decrease as the infection progresses, which has been speculated to be due to the parasite inducing a modulation in the host response to evade its immune system (48). The results of immunohistochemistry for Foxp3 in the analyzed cetaceans also suggested a chronic stage of the disease, which may be indicating a similar evading system in the trematodes of cetaceans to that of *F. hepatica*. Since the infection rate of *C. oblonga* is extremely high in both the harbor and Dall's porpoises of northern Japan by exceeding 95% (excluding calves; unpublished data), obtaining samples from non-infected animals and animals at an early stage of infection is challenging. Further verifications are needed on the correlation between regulatory T cell numbers in uninfected cetaceans and in the initial infection of hepatic trematodiasis.

Hepatocytes in the majority of harbor and Dall's porpoises contained cytoplasmic vacuoles. Cetaceans under highly stressful conditions such as stranding and bycatch respond with various physiological changes (49–51). These changes include acute hepatic congestion due to alterations in the blood flow and secondary hypoxic hepatocellular injury, possibly leading to formations of hyaline inclusions in hepatocytes (13). The cytoplasmic vacuoles observed in the hepatocytes of the current harbor and Dall's porpoises were likely to be caused by the same mechanism and hence are not directly related to trematode infections.

Many of the trematodes of Brachycladiidae are yet to be genetically sequenced. Even for species such as *C. oblonga* (36, 38) and *O. macrorchis* (26) that do have their partial sequences available, there is much uncertainty about the intraspecies mutations and regional variations. Regarding the genetics of *C. oblonga*, the current study provided information of this species outside Europe (Baltic Sea and North Sea) for the first time. Differences of up to 21 bp out of 344 bp in the mtND3 region were identified between the waters off Hokkaido and Europe, indicating regional genetic variations in *C. oblonga*, analogous to regional variations in the harbor porpoises themselves (52). Moreover, one genotype of *C. oblonga* was found in both the harbor and Dall's porpoises around Hokkaido, suggesting coevolution of *C. oblonga* and the two porpoise species.

Investigations on the distribution of hepatic macrophages and cellular responses to various diseases in wildlife, including cetaceans, are still an area with limited research activities (53). Hepatic trematodiasis is a concern not only in wild cetaceans but also in captive animals (54) that requires treatment. This study concludes that there are interspecies differences in tissue reactions to hepatic trematode infections among different odontocete species and that the immune reactions also vary depending on the species. The obtained information aids in furthering our understanding of the pathogenesis of hepatic trematodiasis in cetaceans and its implications to the host. Furthermore, the distinct immune responses noted among the three species will serve as baseline knowledge when assessing the pathology of different diseases among odontocetes.

## Data Availability Statement

The datasets generated for this study can be found in the GenBank/DDBJ database under accession nos. LC532144–LC532151.

## Ethics Statement

Ethical review and approval was not required for the animal study because no live animals were used in this study and all animals were found dead.

## Author Contributions

SN conceived, designed, carried out the experiments, and drafted the manuscript. YK supervised the study, gave conceptual advice, and revised the manuscript. All authors analyzed the data.

## Conflict of Interest

The authors declare that the research was conducted in the absence of any commercial or financial relationships that could be construed as a potential conflict of interest.
