# Supplementary material for: Histochemical and Immunohistochemical Characterizations of Hepatic Trematodiasis in Odontocetes
Source: Front Vet Sci. 2020 Jun 30;7:336. doi: 10.3389/fvets.2020.00336 (PMC7344244; doi:10.3389/fvets.2020.00336)
Supplement: Supplementary file 1 [file Data_Sheet_1.docx]

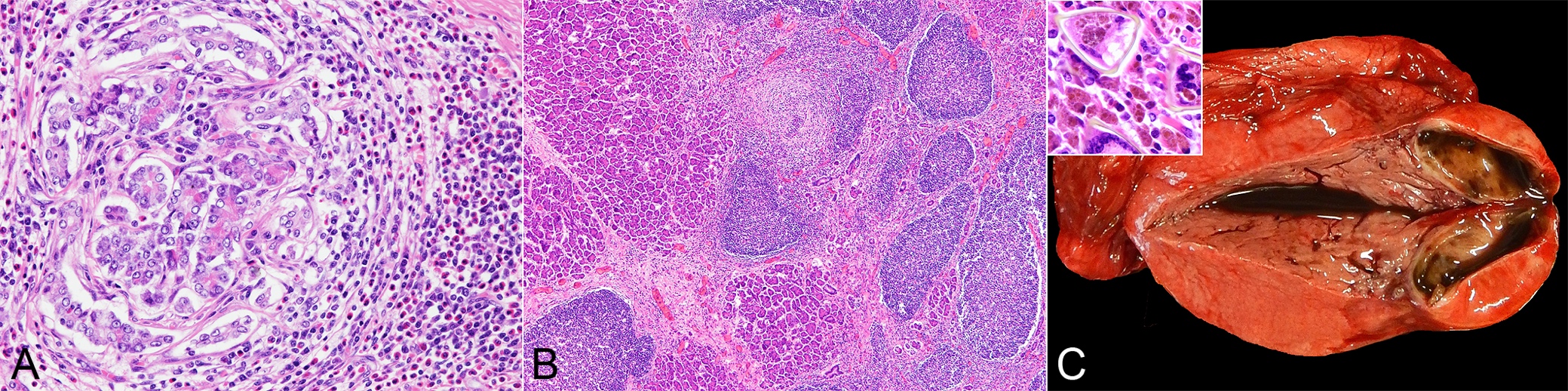


**Supplementary Figure 1 |** *Campula oblonga*-induced pathology, pancreas, harbor and Dall’s porpoises. (**A**) Severe eosinophil-rich inflammation in an area adjacent to the parasitized pancreatic duct. Note the inflammatory cells infiltrating into and effacing the pancreatic parenchyma. Case no. 8, harbor porpoise. (**B**) Variably sized lymphoid follicles are formed not only in the interlobular connective tissue but also frequently within the pancreatic parenchyma. Case no. 15, Dall’s porpoise. (**C**) Gross view of a granulomatous cyst in the pancreas, which contains dark green, mucoid material. Inset, histopathology of the cyst wall reveals abundant inflammatory cells primarily of pigment-laden macrophages, with intralesional triangular-shaped eggs. Case no. 9, Dall’s porpoise.
